# Spontaneous Remission of Metastatic Castration-Resistant Prostate Cancer: Coley’s Toxin Revisited?

**DOI:** 10.7759/cureus.32505

**Published:** 2022-12-14

**Authors:** Albert Choe, Adam Mutsaers, George Rodrigues, Joseph Chin, Stephanie Leung, Eric Winquist

**Affiliations:** 1 Schulich School of Medicine & Dentistry, Western University, London, CAN; 2 Division of Radiation Oncology, Department of Oncology, Western University, London, CAN; 3 Division of Urology, Department of Surgery, Western University, London, CAN; 4 Division of Body Imaging, Department of Medical Imaging, Western University, London, CAN; 5 Division of Medical Oncology, Department of Oncology, Western University, London, CAN

**Keywords:** psa response, metastatic castration resistant prostate cancer, coley’s toxin, immunotherapy, spontaneous remission

## Abstract

Metastatic castration-resistant prostate cancer (mCRPC) is an incurable disease associated with poor survival outcomes. Immunotherapy was first pioneered by William Coley in the early 20th century with the injection of live and heat-killed bacteria. Despite the recent emergence of cancer immunotherapy, mCRPC remains an elusive immune target. Spontaneous remission of mCRPC following microbial infection has not been described in the literature to date. We present evidence of spontaneous biochemical and radiologic regression in a patient with mCRPC following multiple episodes of sepsis.

## Introduction

Prostate cancer is the most common cancer in males and is responsible for approximately 416,000 deaths globally each year [1]. When treated in its early stages, prostate cancer can often be cured; however, recurrent, metastatic prostate cancer is incurable. Metastatic castration-resistant prostate cancer (mCRPC) is a common pathway to prostate cancer death and is associated with a median overall survival of fewer than three years [2]. The treatment landscape for mCRPC is rapidly evolving. The emergence of novel antiandrogen and genomically targeted therapies has significantly improved survival and quality of life [3,4]. Immunotherapy has been rapidly adopted for the treatment of many solid cancers; however, trials of immune checkpoint inhibitors have been disappointing in mCRPC [5]. Currently, sipuleucel-T is the only FDA-approved immunotherapy for mCRPC [6]. Manipulation of the immune system to control cancer is not a novel concept. In the early 20th century, William Coley, an orthopedic surgeon, treated bone sarcomas using a mixture of live and heat-killed bacteria known as “Coley’s toxin” [7]. We report a case of spontaneous remission of mCRPC, which, we hypothesize, occurred due to repeated episodes of bacteremia.

## Case presentation

An 80-year-old white male underwent treatment with a radical prostatectomy for pT3, Gleason 7 (4+3) adenocarcinoma of the prostate 14 years ago. His other relevant past medical history included distant myocardial infarction, type 2 diabetes mellitus treated with metformin, and stage III colon adenocarcinoma treated with right hemicolectomy and adjuvant FOLFOX (fluorouracil, leucovorin, and oxaliplatin) chemotherapy 11 years prior. The serum prostate-specific antigen (PSA) was elevated post-prostatectomy and the patient received salvage external beam radiation therapy and androgen-deprivation therapy (ADT) with depot goserelin injections.

**Figure 1:**
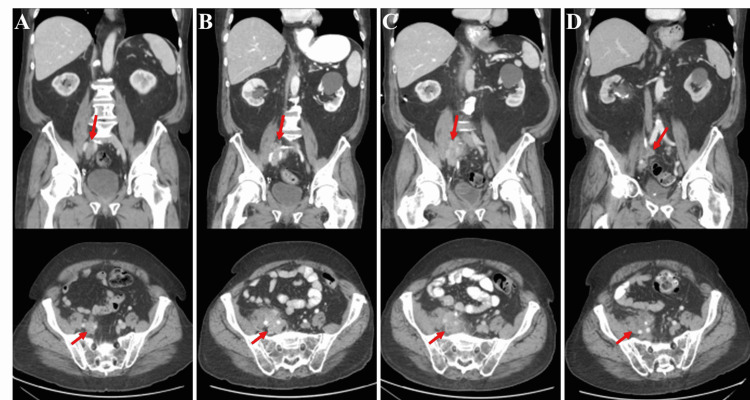
Serial CT of the abdomen and pelvis demonstrating interval changes in tumor burden in December 2016, January 2018, May 2018, and September 2019 (left to right) (A) Ill-defined lobular soft tissue mass (1.7 x 1.3 cm) below the bifurcation of the right common iliac vessel. (B) Interval increase in soft tissue mass size, measuring 4.3 x 3.5 cm. (C) Slightly reduced soft tissue mass size (4.1 x 3.5 cm). (D) Interval increase in the size of lobulated soft tissue mass (4.9 x 3.7 cm), now encasing distal right ureter CT: computed tomography

The patient was diagnosed with non-metastatic CRPC in August 2015 based on the observation of rising PSA levels on ADT. He received apalutamide on a clinical trial but discontinued the therapy after four months due to nausea and diarrhea. He was observed on ADT and subsequently developed right leg swelling in December 2016. A CT scan identified a mild right hydroureter due to a 1.7 x 1.3-cm lobulated soft tissue mass at the bifurcation of the right common iliac vessel (Figure 1A), which was presumed to be malignant. The scan also revealed deep venous thrombosis and he was treated with dalteparin. In April 2017, abiraterone plus prednisone was started; however, PSA levels continued to rise. Treatment was switched to enzalutamide in July 2017 with a PSA decline from 20.59 to 5.07 μg/L.

The patient experienced nausea and diarrhea with enzalutamide, necessitating dose reduction, and by January 2018, the PSA had increased to 29.77 μg/L. The CT scan showed an enlargement of the right pelvic mass to 4.3 x 3.5 cm (Figure 1B). Enzalutamide was discontinued, and a right-sided nephrostomy tube was inserted. A 15-fraction course of palliative radiation to the pelvic mass was planned; however, after the first fraction of 200 cGy radiation, the patient became ill with fever, rigors, diarrhea, and nausea and was admitted to the hospital. No further radiation was delivered. The patient was diagnosed with urosepsis and *Klebsiella pneumoniae *bacteremia and treated with broad-spectrum antibiotics. Two months later, the patient was re-admitted with *Pseudomonas aeruginosa *bacteremia with a urinary source considered likely.

**Figure 2:**
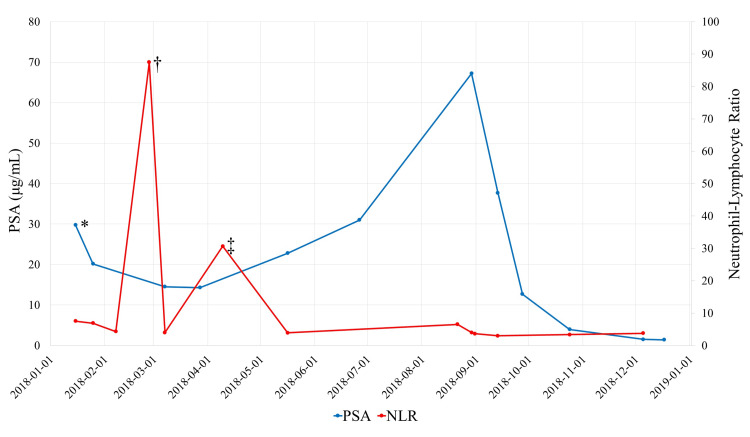
PSA and NLR trends over the course of two bacteremia episodes Bacteremia episodes occurred in February 2018 (†) and April 2018 (‡). Enzalutamide was last administered in January 2018 (*) PSA: prostate-specific antigen; NLR: neutrophil-to-lymphocyte ratio

The patient was observed on depot goserelin alone, and PSA levels rose, peaking at 67.20 μg/L in August 2018. One month later, the PSA level dropped to 12.70 μg/L and continued to fall without any alteration in therapy (Figure 2).

At this time, concomitant medications included atorvastatin, metformin, hydromorphone, fesoterodine, omeprazole, ramipril, rivaroxaban, venlafaxine, ranitidine, and depot goserelin. The patient denied the use of antiandrogens, estrogens, megestrol acetate, supplements, naturopathic interventions, or significant diet or lifestyle changes. Serum testosterone remained suppressed since the initiation of ADT.

CT scan in May 2018 showed a slightly reduced pelvic mass of 4.1 x 3.5 cm (Figure 1C) and the presence of new aortocaval and right common iliac lymphadenopathy. By December 2018, the PSA reached a nadir of 1.40 μg/L. PSA began rising again in March 2019 and CT in September 2019 showed an interval increase of the right lobulated mass to 4.9 x 3.7 cm (Figure 1D) and new soft tissue deposits along the right external iliac vessels. Conversely, the retroperitoneal lymphadenopathy had improved since the previous year, suggesting reactive lymphadenopathy rather than tumor burden. After a final CT scan in March 2020 demonstrated progressive tumor burden, the patient declined further cancer treatment and pursued medical assistance in dying in June 2020.

## Discussion

Spontaneous remissions of mCRPC are rare. Withdrawal of antiandrogens is known to induce PSA response in some patients [8-10]. Our patient had a minor transient drop in PSA after discontinuing enzalutamide (29.77 to 15.50 μg/L) but showed unequivocal disease progression. We do not believe this phenomenon explains the more dramatic and subsequently sustained remission.

Metformin is known to be associated with PSA response. Two of 38 patients (5.3%) with mCRPC had a PSA drop of over 50% with metformin monotherapy in a phase-2 clinical trial [11]. However, the dose of metformin administered to our patient was maintained for over a decade, so we consider the effect of this drug unlikely in this case.

The neutrophil-to-lymphocyte ratio (NLR) is a non-specific marker of systemic inflammation and is correlated with poor prognosis in prostate cancer [12]. In our patient, each septic episode was associated with a peak in NLR (Figure 2), and these episodes were followed by a rapid rise and dramatic fall in PSA without new therapy.

William Coley first injected a cancer patient with streptococcal organisms in 1891 based on observation of tumor regression in a patient with erysipelas, along with anecdotal reports of tumor regression associated with infection in the medical literature of the time [7]. He observed tumor responses and treated nearly 1000 patients with his eponymous toxin consisting of heat-killed streptococcal bacteria combined with an organism we now refer to as *Serratia marcescens*. Parke Davis and Company marketed a toxin based on his work for 30 years starting in 1899; however, the use of Coley’s toxin fell out of favor with the emergence of radiation therapy. Decades later, the bacillus of Calmette and Guérin (BCG) emerged as an early example of immunotherapy that was made from a live, attenuated strain of *Mycobacterium bovis *and remains a standard treatment for non-muscle-invasive bladder cancer [13]. Although Coley was criticized during his own lifetime, he is now considered by many to be the “Father of Immunotherapy”.

Our patient appeared to have a spontaneous biochemical and radiological response with marked, sustained PSA drop (67.20 to 1.40 μg/L) and a slightly smaller pelvic mass in May 2018 (Figure 1C). However, this occurred after two serious episodes of Gram-negative bacteremia requiring hospitalization. Coley’s toxin included an encapsulated Gram-negative bacterium. Also, the first episode of bacteremia was coincident with a single, low dose of palliative radiation. Low-dose radiation may enhance the anti-tumor immune response by inducing major histocompatibility complex I upregulation, chemokines, and cytokines that promote immune cell infiltration and activation, and increased expression of tumoral neoantigens [14]. The radiation dose received by this patient was very low but coincident with septicemia, which makes us wonder if this exposure may have also contributed to an immune response.

Prostate cancer is not considered responsive to immunotherapy. Antonarakis et al. reported objective response in 3-5% of mCRPC patients treated with pembrolizumab in a large phase-2 trial [5]. Our case may illustrate the potential sensitivity of prostate cancer to the adaptive immune system. Meanwhile, the research to identify predictive biomarkers of response to immunotherapy and to develop more active forms of immunotherapy for prostate cancer continues apace.

## Conclusions

We discussed the case of an mCRPC patient with spontaneous biochemical and radiologic remission following two episodes of Gram-negative bacteremia. We considered the effects of anti-androgen withdrawal, use of metformin, and one-time use of palliative radiation therapy on tumor burden; however, we ultimately hypothesize an immune-mediated effect. This case reinforces the idea that mCRPC can be immune-responsive and supports the continuing studies on immunotherapy in prostate cancer.
